# Épidémiologie et facteurs de risque de l'hypertension artérielle persistante au décours d'une pré-éclampsie: étude longitudinale à l'Hôpital de l'Amitié Tchad-Chine

**DOI:** 10.11604/pamj.2024.49.44.43783

**Published:** 2024-10-18

**Authors:** Mianroh Hybi Langtar, Adjougoulta Koboy Allah-Amine, Dounè Narcisse, Maimouna Djibrine Kiram, Naibe Dangwe Tamoua, Abakar Bachar, Mbessoh Kengne Ulrich Igor, Mahamat Alhadji Chene, Idriss Daraiya Alsimbilaya

**Affiliations:** 1 Service de Cardiologie de l'Hôpital de l'Amitié Tchad-Chine, Ndjamena, Tchad,; 2 Service de Cardiologie, Centre Hospitalier Universitaire la Référence Nationale, Ndjamena, Tchad,; 3 Service de Cardiologie, Centre Hospitalier Universitaire la Renaissance, Ndjamena, Tchad,; 4 Université Cheik Anta Diop, Dakar, Sénégal, Ndjamena, Tchad; 5 Service de Gynéco-Obstérique de l'Hôpital de l'Amitié Tchad-Chine, Ndjamena, Tchad

**Keywords:** Prééclampsie, HTA persistante, Tchad, preeclampsia, persistent hypertension, Chad

## Abstract

**Introduction:**

l'hypertension artérielle (HTA) peut persister au-delà de 03 mois du post-partum après une pré-éclampsie. Elle augmente le risque de complications cardiovasculaires sur le long terme. Notre objectif est de décrire les aspects épidémiologiques et les facteurs associés à la persistance de l'hypertension artérielle au décours d'une pré-éclampsie.

**Méthodes:**

il s'agissait d'une étude descriptive longitudinale, allant de janvier 2022 à juin 2023. Les patientes hospitalisées pour une pré-éclampsie à la maternité de l'Hôpital de l'Amitié Tchad-Chine et suivies en consultation externe de cardiologie pendant au moins 03 mois étaient incluses. Nous avons effectué une analyse multivariée par régression logistique binaire pour identifier les facteurs associés à l'hypertension persistante.

**Résultats:**

durant notre période d'étude, 144 patientes étaient hospitalisées pour pré-éclampsie dont 92 étaient incluses. L'âge moyen des patientes était de 26,32±7,05 ans avec des extrêmes allant de 16 à 42 ans. Environ 41,3% (n=38) étaient des multipares, la pré-éclampsie était sévère chez 80,4% (n=74), 17,4% (n=16) avaient une grossesse gémellaire, l'obésité était retrouvée chez 19,6% (n=18). L'hypertension artérielle était persistante chez 24 soit une prévalence de 26,10% (n=24), l'antécédent personnel de pré-éclampsie était le seul facteur associé à l'hypertension artérielle persistante retrouvé ORa 5,30 IC 95% 1,31-21,44 p=0,01.

**Conclusion:**

il serait nécessaire de mettre en place un parcours de soins des patientes qui avaient fait une pré-éclampsie afin de prévenir et de prendre en charge précocement les complications au long cours.

## Abstract (en)

**Introduction:**

arterial hypertension (AH) may persist beyond three months postpartum after preeclampsia, increasing the long-term risk of cardiovascular complications. The purpose of this study is to describe the epidemiological aspects and factors associated with persistent hypertension following preeclampsia.

**Methods:**

we conducted a longitudinal descriptive study, from January 2022 to June 2023. The study included patients who were hospitalized for preeclampsia in the maternity ward of the Chad-China Friendship Hospital and followed in the cardiology outpatient clinic for at least three months. A multivariate analysis using binary logistic regression was performed to identify factors associated with persistent hypertension.

**Results:**

during our study period, 144 patients were hospitalized for preeclampsia, of whom 92 were included in the study. The average age of patients was 26.32 ±7.05 years, with extremes ranging from 16 to 42 years. Multiparity was observed in 41.3% (n=38) of patients and severe preeclampsia occurred in 80.4% (n=74). Additionally, 17.4% (n=16) had twin pregnancies and obesity was found in 19.6% (n=18). Persistent arterial hypertension was present in 24 patients, representing a prevalence of 26.10% (n=24). A personal history of preeclampsia was the only factor significantly associated with persistent arterial hypertension (adjusted OR 5.30, 95% CI 1.31-21.44, p=0.01).

**Conclusion:**

it is necessary to develop a care pathway for patients who have had preeclampsia in order to prevent and manage long-term complications at an early stage.

## Introduction

La pré-éclampsie se définit cliniquement par l'association d'une hypertension artérielle gravidique (PAS ≥ 140 mmHg et/ou PAD ≥ 90 mmHg) et d'une protéinurie massive > 300 mg/24h survenant à partir de la 20^e^ semaine d'aménorrhée (SA) [1]. Elle représente l'une des premières causes de mortalité materno-fœtale à l'échelle mondiale, elle touche environ 4 à 5% des grossesses dans le monde [2]. Cette pathologie qui est devenue moins fréquente dans les pays développés reste encore d'actualité en Afrique subsaharienne [3,4]. Selon une revue systématique et une méta analyse réalisée de 1997 à 2007 en Afrique la prévalence de la pré-éclampsie (PE) était estimée à 5,3% [5].

Au Tchad sa prévalence était de 2,9% en 2019 [6]. Après une pré-éclampsie, la tension artérielle se normalise habituellement au bout de 06 semaines [7]. Malheureusement chez certaines patientes elle reste élevée au-delà de cette période. L'hypertension artérielle (HTA) est dite persistante au décours d'une pré-éclampsie lorsque la tension artérielle (TA) ne se normalise pas après 3 mois du post-partum [8]. Elle augmente chez les jeunes mamans le risque de développer des complications cardiovasculaires liées à l'HTA. En Afrique, les études menées par Lugobe *et al*. en Ouganda et Amougou *et al*. au Cameroun ont retrouvé une prévalence respective de 23% et 39% de l'HTA persistante [8,9]. Dans notre contexte nous n'avons pas des études rapportant les données sur l'HTA persistante. Le suivi en post-partum des patientes après une pré-éclampsie n'est pas bien codifié cela entraine une perte de vue importante des patientes qui vont développer leur HTA sans une prise en charge adéquate. Ainsi l'objectif de notre travail est d'étudier les aspects épidémiologiques et rechercher les facteurs prédisposant à développer une HTA persistante afin d'améliorer le suivi et la prise en charge de ces patientes au long cours.

## Méthodes

**Conception et cadre de l'étude:** il s'agissait d'une étude descriptive longitudinale réalisée dans le service de gynéco-obstétrique et de cardiologie de l'Hôpital de l'Amitié Tchad-Chine de janvier 2022 à Juin 2023 Soit une période de 18 mois. L'hôpital de l'Amitié Tchad-Chine est un hôpital public de niveau 3 selon l'organigramme du Ministère de la Santé Publique. Il est situé en pleine capital Ndjamena (Tchad).

**Population d'étude:** notre étude concernait toutes les patientes hospitalisées pour une pré-éclampsie dans le service de gynécologie obstétrique, cependant celles qui étaient suivies en post-partum à la consultation externe de cardiologie pendant au moins 03 mois étaient incluses. Toutes les patientes non consentantes, celles perdues de vue et celles ayant un antécédent d'HTA étaient exclues. L'échantillonnage était fait de manière consécutive non exhaustive.

**Collecte de données:** les données étaient collectées pendant l'hospitalisation et lors des consultations externes en post-partum à l'aide d'une fiche préétablie. Les patientes étaient vues en consultation externe de cardiologie avant leur sortie de l'hôpital où la tension artérielle était prise aux 2 bras après un repos d'au moins 5 minutes par un tensiomètre de marque Omron M6 Comfort. Un interrogatoire et un examen clinique étaient réalisés, puis elles étaient revues tous les mois pendant au moins 3 mois.

**Définitions:** les variables étudiées étaient sociodémographiques (âge, résidence, niveau d'instruction), cliniques (TA, Indice de masse corporelle, antécédents) et les facteurs prédisposant. L'HTA persistante était définit comme TA supérieure ou égale à 140 mmHg pour la pression artérielle systolique (PAS) et/ou 90 mmHg pour la pression artérielle diastolique (PAD) ou une tension artérielle normale sous traitement au-delà de 3 mois du post-partum. La pré-éclampsie était définit par l'association d'une hypertension artérielle gravidique (PAS ≥140 mmHg et/ou PAD ≥90 mmHg) et d'une protéinurie massive >300 mg/24h survenant à partir de la 20^e^ semaine d'aménorrhée (SA). Elle était sévère lorsqu'elle associe une HTA sévère (PAS ≥160 mmHg et/ou PAD ≥110 mmHg) et une protéinurie ≥5 g/24 heures. Une primipare était définie comme une femme qui a accouché pour la première fois, une multipare était définie comme une femme qui a accouché au moins quatre fois. Entre les deux on parle de paucipare. L'obésité est définie par un indice de masse corporel supérieur à 30 kg/m^2^, le surpoids est défini par indice de masse corporel entre 25 et 30 kg/m^2^.

**Analyses statistiques:** les données étaient saisies et analysées grâce au logiciel SPSS. Les variables qualitatives étaient exprimées en pourcentage et les variables quantitatives en moyenne avec leurs écarts type. Le test statistique Khi carré était utilisé pour faire la comparaison entre les variables avec un seuil de significativité dit positif lorsque P < 0,05. Nous avons effectué une analyse bivariée pour rechercher les probables facteurs prédisposants, puis une analyse multivariée par régression logistique binaire pour confirmer l'existence d'une association entre les facteurs prédisposants et la persistance de l'HTA, ainsi que supprimer les facteurs de confusion.

**Considérations éthiques:** nous avons reçu le consentement éclairé des patientes ainsi que l'accord du comité éthique de l'Hôpital de l'Amitié Tchad Chine.

## Résultats

**Figure 1:**
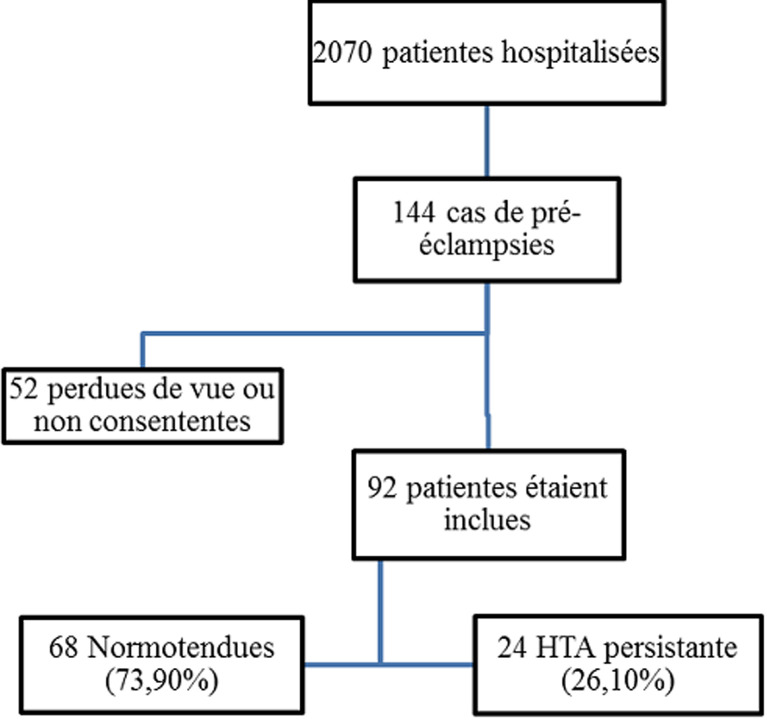
représentation de la prévalence de l'HTA persistante

**Prévalence:** durant notre période d'étude, 2070 patientes étaient hospitalisées dans le service de gynéco-obstétrique. Cependant, 144 patientes ont présenté une pré-éclampsie parmi lesquelles 92 étaient incluses et 52 étaient exclues (Perdues de vue ou non consentantes). L'hypertension artérielle était persistante chez 24 soit une prévalence de 26,4% (Figure 1).

**Données sociodémographiques:** l'âge moyen de nos patientes était de 26,32±7,05 avec des extrêmes allant de 16 à 42 ans. Environ 73,9% (n=68) des patientes étaient des ménagères, 32,6% (n=30) étaient non instruites, la majorité vivait en milieu urbain soit 89,1% (n=82) (Tableau 1).

**Tableau 1:** données sociodémographiques des patientes

	Nombre (n)	Fréquence (%)
**Niveau d'étude**		
Primaire	17	18,5
Secondaire	34	36
Universitaire	11	12
Non instruite	30	32,6
**Résidence**		
Urbain	82	89,1
Rural	10	10,9
**Profession**		
Ménagère	68	73,9
Commerçante	20	21,7
Fonctionnaire	4	4,3
**Age**		
< 20 ans	31	33,7
21-25	15	16,3
26-30	25	27,2
> 30	21	22,8

**Données cliniques:** l'antécédent personnel de pré-éclampsie était retrouvé chez 10,9% (n=10), 41,3% (n=38) étaient des multipares, les primipares représentaient 39,1% (n=36). La majorité des patientes avait une grossesse monofoetale 82,6% (n=76), la grossesse était gémellaire chez 17,4% (n=16). 19,6% (n=18) des patientes étaient obèses et 26,1 (n=24) en surpoids. Plus de la moitié des patientes ont présenté une pré-éclampsie sévère 80,4% (n=74). Les céphalées étaient le signe fonctionnel le plus ressentis soit 71,7% (n=66).

**Facteurs prédisposants:** dans notre série, les facteurs prédisposants à la survenue de l'HTA persistante recherchés en analyse bivariée étaient l'antécédent familial d'HTA (P=0,99) et de pré-éclampsie (P=0,25), l'antécédent personnel de pré-éclampsie (P=0,02), le surpoids (P=0,91) et l'obésité (P=0,52), la primiparité (P=0,61) et la multiparité (P=0,97), la gravité de la pré-éclampsie sévère (P=0,73) et légère (P=0,89), grossesse gémellaire (P=0,53) et grossesse unique (P=0,82). L'antécédent personnel de pré-éclampsie était le seul facteur prédisposant à la survenue de l'HTA persistante retrouvé en analyse multivariée par régression logistique binaire ORa 5,30 IC 95% 1,31-21,44 p=0,01 (Tableau 2).

**Tableau 2:** facteurs prédisposant à la survenue de l'HTA persistante

	Normotendue n(%)	Hypertendue n(%)	P-value	ORa (IC 95%)	P-value
**Antécédent personnel**					
pré-éclampsie	4 (5,9)	6 (25,0)	0,027	5,30(1,31-21,44)	0,01
**Parité**					
Primipare	25 (36,8)	11 (45,8)	0,61	0,42(0,09-1,89)	0,26
Multipare	28 (41,2)	10 (41,7)	0,97	0,53(0,11-2,39)	0,41
Paussipare	15 (22,1)	3 (12,5)	0,39	1,96(0,51-7,55)	0,32
**Gravité de pré-éclampsie**					
Pré-éclampsie légère	14 (20,6)	4 (16,7)	0,89	1,38(0,40-4,78)	0,60
Pré-éclampsie sévère	54 (79,4)	20 (83,3)	0,73	0,85(0,23-3,09)	0,80
**Gémellité**					
Grossesse gémellaire	13 (19,1)	3 (12,5)	0,53	1,44(0,35-5,98)	0,61
Grossesse unique	55 (80,9)	21 (87,5)	0,82	0,60(0,15-2,37)	0,47

## Discussion

Notre étude a permis de faire le suivi en post-partum pendant au moins 3 mois en consultation externe de cardiologie des patientes qui étaient hospitalisées pour une pré-éclampsie. L'objectif était de décrire les aspects épidémiologiques de l'hypertension artérielle persistante et de rechercher les facteurs prédisposants à sa survenue. L'hypertension artérielle persistait chez 26,1% (n=24), le facteur de risque retrouvé dans notre contexte était l'antécédent personnel de la pré-éclampsie ORa 5,30 IC 95% 1,31-21,44 p=0,01.

En post-partum, après une pré-éclampsie la tension artérielle se normalise en moyenne après 06 semaines, chez 75% de ces patientes la tension artérielle se stabilise après 2 mois et chez 90% après 3 mois [10]. Nous avons suivi nos patientes pendant au moins 3 mois et il en ressort une prévalence de 26,1% de l'hypertension artérielle persistante. D'où l'importance d'un suivi en post-partum des patientes après une pré-éclampsie afin de diagnostiquer et prendre en charge tôt ces patientes pour éviter les complications au long cours. En l'absence d'un suivi adéquate, l'HTA persistante peut passer inaperçue augmentant ainsi le risque de complications cardiovasculaires et d'insuffisance rénale à long terme [11-14]. Ce qui constitue un lourd fardeau, surtout dans notre contexte où les patientes se prennent en charge elle-même ou par leur famille. Nos résultats sont comparables à ceux d'Amougou *et al*. [9] au Cameroun en 2019, Raymond *et al*. [15] en France en 2017 qui trouvaient une prévalence respective de 23,5% et 27%. Lugobe *et al*. [8] en Ouganda en 2019 quant à lui trouvait une prévalence de l'HTA persistante de 39%. Cette différence peut s'expliquer par sa population d'étude qui était constituée des patientes ayant présenté une pré-éclampsie, une HTA gravidique et une éclampsie. Le mécanisme de survenue de l'HTA persistante n'est pas très bien élucidé. Cependant, les études récentes montraient que la pré-éclampsie et les maladies cardiovasculaires partagent les mêmes facteurs de risque, notamment les troubles métaboliques, des troubles inflammatoires, le stress oxydatif et l'hypercoagulabilité [3]. Plusieurs études observationnelles démontraient qu'il existe un risque 2,28 fois plus élevé de futures maladies cardiovasculaires chez les femmes qui ont eu une pré-éclampsie [16]. Par conséquent, il serait judicieux d'établir un calendrier de consultation adéquat et précis pour ces patientes en post-partum.

Nous avons aussi suivi nos patientes afin de déterminer d'éventuels facteurs de risque prédisposant à la persistance de l'hypertension artérielle. Les facteurs de risque recherchés étaient la parité, la grossesse gémellaire, l'antécédent personnel de pré-éclampsie, et la sévérité de la pré-éclampsie. Après analyse multivariée, l'antécédent personnel de pré-éclampsie était le seul facteur de risque de survenue de l'hypertension artérielle persistante retrouvé dans notre série (ORa 5,30 IC 95% 1,31-21,44 p=0,01). Il a été remarqué que les femmes ayant un antécédent de pré-éclampsie avaient un risque 2 fois plus élevé de développer un accident vasculaire cérébral, une arythmie, une insuffisance cardiaque et le risque de développer une insuffisance rénale était multiplié par 10 chez ces dernières [10]. La période du post-partum constitue le véritable moment d'identification de ces patientes à risque. Bokslag *et al*. avaient fait le même constat, ils avaient retrouvé dans leur série un risque plus élevé de développer une hypertension artérielle chez les patientes ayant un antécédent de pré-éclampsie précoce (avant 34 SA) [17]. Pendant la première année ce risque était relativement un peu plus faible 22% selon les études réalisées par Van *et al*. [18]. Drost *et al*. trouvaient que ce risque augmentait au fil des années chez ces patientes avec une prévalence d'HTA qui pouvait atteindre 43% 9 ans après un premier épisode de pré-éclampsie [19]. Mito *et al*. avaient remarqué dans leur étude que les femmes ayant un antécédent de pré-éclampsie avaient un risque plus élevé de développer une HTA chronique 5 ans après la grossesse de référence (OR 7.1; 95% CI [2.0-25.6]) [20]. A cela s'associe un risque accru de développer des pathologies cardiovasculaires. En plus d'un suivi précoce, les femmes avec un antécédent de pré-éclampsie doivent être informées de la nécessité de se faire suivre au long cours. Cependant la période, l'intervalle de suivi reste à déterminer car il n'existe pas encore de consensus. Néanmoins un contrôle régulier de la tension artérielle à domicile par les patientes peut être proposé comme alternative permettant de suivre l'évolution de leur tension artérielle et se faire consulter en cas de persistance ou d'augmentation des chiffres tensionnels.

Dans notre contexte, la majorité des femmes se rendent à l'hôpital que lorsqu'elles sont malades. Selon un essai randomisé contrôlé multicentrique, la surveillance de la tension artérielle à domicile chez les femmes ayant un antécédent de pré-éclampsie pendant un an permettait d'augmenter le nombre de diagnostics d'hypertension (34% dans le groupe d'intervention contre 11% dans le groupe témoin p<0.001) [21]. L'*American Heart Association* reconnait l'antécédent de pré-éclampsie comme un facteur de risque des maladies cardiovasculaires spécifique aux femmes indépendamment de l'âge et l'origine ethnique [22,23]. Elle souligne aussi le fait que la période du post-partum pourrait être le moment idéal de dépistage des facteurs de risque cardiovasculaires afin de prévenir les complications ultérieures.

D'autres études sur une longue période seraient nécessaires dans notre contexte pour mieux évaluer l'hypertension artérielle chez les patientes qui avaient présenté une pré-éclampsie. Le nombre important des patientes perdues de vue et non consentantes (52 patientes) a certainement impacté nos résultats. Nous n'avons pas utilisé le Holter tensionnel des 24 heures, ce qui n'a pas permis de mettre en évidence les patientes qui présentaient une HTA blouse blanche ou une HTA masquée.

## Conclusion

L'hypertension artérielle persistante après une pré-éclampsie est une pathologie relativement fréquente dans notre contexte mais elle est malheureusement sous diagnostiquée. Un accent particulier doit être mis surtout sur celles qui avaient un antécédent personnel de cette pathologie. Ainsi le suivi en post-partum des patientes qui avaient présenté une pré-éclampsie doit être obligatoire pendant au minimum 3 mois afin de dépister et d'anticiper sur la prise en charge des facteurs de risque cardiovasculaires.

### Etat des connaissances sur le sujet

- L’HTA peut persister chez certaines femmes au décours de la pré-éclampsie au-delà de 03 mois;
- Plusieurs facteurs de risque prédisposent à la survenue de l'HTA persistante;
- Le suivi en post-partum des patientes après une pré-éclampsie n'est pas bien codifié.

### Contribution de notre étude à la connaissance

- Notre étude a permis de déterminer la prévalence hospitalière de HTA persistante au décours d'une pré-éclampsie dans notre contexte;
- Notre étude a également permis de structurer le suivi des patientes après une pré-éclampsie;
- La mise en évidence de l'antécédent de pré-éclampsie comme facteur prédisposant à la survenue de l'HTA persistante.

## Remerciements

Nous remercions toutes les patientes qui ont accepté de participer à l'étude ainsi que le personnel médical et paramédical du service de gynécologie et de cardiologie de l'Hôpital de l'Amitié Tchad-Chine.
